# Economic evaluation of process utility: elucidating preferences for a non-invasive procedure to treat restenosis

**DOI:** 10.1186/s13561-021-00327-x

**Published:** 2021-07-23

**Authors:** Maria V. Aviles-Blanco

**Affiliations:** Department of Financial Economics and Operations Management, Faculty of Economics and Business Administration, University of Sevilla, Avda. Ramón y Cajal,1, 41018 Sevilla, Spain

**Keywords:** Process utility, Willingness to pay, QALY, Angioplasty, H41, H51, I11, I18, I31

## Abstract

**Background:**

In health economic evaluation, utility associated with a health state is outcome-oriented and usually measured using the QALY methodology. Even though there is consistent evidence of utility not only being derived from outcomes but also from procedures, process utility has not been fully integrated in QALY calculations. The aim of this paper is twofold: first, to provide evidence of process utility associated with an alternative treatment to angioplasty, and second, to estimate a monetary value of such process utility using the willingness to pay (WTP) approach.

**Methods:**

A total of 1514 people were polled on their WTP to avoid angioplasty to have a drug-eluting stent (DES) implanted. WTP is estimated with a contingent valuation (CV) survey. Individuals are also asked if they would be WTP for a non-invasive procedure with similar results being achieved. WTP responses were analyzed using a double bounded (DB) logit model.

**Results:**

Most of the participants showed positive preferences for avoiding angioplasty, with an estimated mean WTP of €5692.87. Using QALY gains for avoiding angioplasty, varying from 0.0035 to 0.08 QALYs, our WTP estimate imply monetary values per QALY that range from €71,160.87 to €1,626,534.28.

**Discussion:**

A WTP of €5692.87 to avoid angioplasty imply a monetary value per QALY that greatly exceed the cost per QALY thresholds established in different countries to consider health programs as beneficial to society. Our results reflect how different methodologies for HTA may lead to different conclusions. From the ICER perspective, the cost that would make the treatment with pills option cost-effective, using a threshold of €40,000/QALY, would be €224. However, a cost-benefit approach could support health programs even with a higher cost.

**Conclusion:**

WTP methodology captures outcome and process factors related to angioplasty as our WTP estimations are non-significantly different for the costs of angioplasty. WTP approach must be considered as a genuine alternative to QALY approaches to value process utility.

## Background

Utility associated with a health state is often measured in quality-adjusted life years (QALYs).

The QALY approach is considered a consequentialist approach in the sense that use value estimations focus on a final outcome of health gain consequent to health care [1]. Utility associated with a health state is considered to be dependent mainly on outcomes such as duration and severity of a health state [2–9], and measured in the interval *[0, 1],* where one represents full health and zero a state equivalent to death. The QALYs for each health state is then computed multiplying its utility weight by its duration. The gain in QALYs from avoiding an impaired health state is obtained as the difference between QALYs for full health (*H*^*F*^) and QALYs for that health state (*H*^*I*^).

Therefore, it is assumed that health care has no positive value in use and ignores the possibility of having a negative value in use. In case of restenosis, the primary treatment is an intervention to implant a Drug Eluting Stent (DES). However, let us hypothesize with an alternative treatment with pills that would avoid the intervention. There is no pleasantness in any intervention to cure restenosis, all have negative values in use but, to some extent, there might be preferences for one choice over the other. Other things being equal, restenosis is treated in both cases, given a choice with two options individuals value other aspects of procedures and may have preferences for the non-invasive procedure, as in [10–13]. These attributes in process of health care enter the utility function [14, 15], have quality of life implications for the individual [16] and can be incorporated into estimates of QALY calculations [17, 18]. The theoretical background for process utility entering the QALY estimations was first introduced by Gerard and Mooney (1993) [15].

Health outcomes are not the only form of benefit in health care since avoiding discomfort or a sense of fear could affect mental health. Considering process utility as a health gain value would lead to optimal provision of health care. Including process utility as an integral part of Health Technology Assessments (HTAs) would strengthen informative tools for decision makers.

Yet, it is difficult to include process utility values when measuring QALYs. Traditional methods to determine health state utility values (such as standard gamble or time trade-off) might not be efficient in the evaluation of process utility. Since these methods trade health attributes against risk of death or against years of life in full health, the amount of risk one would accept or the amount of time given up for process benefits such as treatment administration [19], testing strategies [16] or caregiver characteristics [20] lead to very low gains in QALYs.

In these cases, in a potential economic evaluation using an incremental cost-effectiveness ratio (ICER), a very low gain in QALYs will need to be offset by a very low cost for a treatment or a test to achieve a cost per QALY lower than the socially accepted cost per QALY. It is, therefore, opportune to analyze alternatives for process utility evaluation that might be incorporated in the evaluation of treatments, screening tests, diagnostic tests or therapies that might end up being underestimated if valuation is obtained with traditional health evaluation methods [20, 21].

The aim of this paper is to explore an alternative for process utility evaluation estimating a monetary value. First, to measure whether process utility exists the study includes a comparison of two treatments for restenosis with identical outcomes and different processes. These treatments for restenosis are A) an angioplasty to implant a Drug-Eluting Stent (DES), and B) a hypothetical treatment with pills. We expect respondents to prefer treatment B over treatment A.

Secondly, WTP is estimated using a contingent valuation (CV) tool. Individuals are asked if they would be willing to pay for a treatment with pills as a substitute for angioplasty, with similar health outcomes being achieved. Our research emphasizes the suitability of WTP as an alternative method to value process utility. Monetary evaluation provides a unique value that incorporates the utility/disutility of perceptions and opportunity costs.

The section here-below describes the sample, the questionnaire and the model used to estimate the mean WTP for the treatment with pills. Section 3 contains the model estimates. Section 4 includes a simulated cost-efficiency and cost-benefit analysis for the treatment with pills option and some concluding observations.

## Methods

To obtain the monetary value of avoiding angioplasty, a CV survey was designed and presented to 1663 individuals. The interviews were conducted at the respondent’s house, using a Computer Assisted Personal Interview (CAPI) methodology, in February–April 2009. The sampling universe was a population resident in Spain of over 19 year old. The survey sample was stratified in seven categories according to habitat size: less than 2000; 2001–10,000; 10,001-50,000; 50,001–100,000; 100,001-400,000; 400,001-1,000,000, more than 1,000,000 residents. The primary sampling units were 108 municipalities representative of the 17 Spanish regions and the secondary sampling units were houses selected along random routes. In-house selection was according to proportions based on gender and age. Respondents voluntarily participated in the survey. Sample population is representative of the Spanish population; socio-demographic characteristics of sample and Spanish populations are shown in Table 1.

**Table 1:** Sample Socioeconomic Characteristics

	Sample population	Spain (2009)
**Age**^**1**^		
*20–34*	29.56	28.3
*35–49*	28.3	29.9
*50–64*	22.5	21.5
*65+*	19.6	20.3
**Gender**^**2**^		
*% female*	50.7	50.6
**Level of studies**^**3**^		
*Compulsory education*	37.9	23.1
*1st level Secondary*	34.5	27.5
*2nd level Secondary*	8.3	21.0
*Higher Education*	19.2	28.5
**Employment**^**4**^		
*Employed*	59.9	60.1
*Unemployed*	40.1	39.8
**Household average size**^**5**^	3.1	2.9
**Net household income**^**6**^		
*Up to 1200€*	39.6	45
*From 1201 to 3000€*	55.3	51.2
*More than 3000€*	5.1	3.8

^**1**,**2**^ Estimations from Census, January 2009. (Padrón Municipal)

^**3**^ Data from 2007 for Spain from the Nacional System of Education Indicators (Sistema estatal de indicadores en educación) http://www.institutodeevaluacion.mec.es/contenidos/pdfs/c4_2007.pdf

^**4**^Employment Survey. (Encuesta de Población Activa) First Quarter 2009

^**5**^Household Budget Survey,(Encuesta de Presupuestos Familiares), 2005

^**6**^Income Survey (Encuesta de Estructura Salarial), 2006

Source: ^1,2,4,5 y 6^: National Institute of Statistics. INE (http://www.ine.es)

### Survey design

The questionnaire was designed to minimise any possible bias or inconsistencies in the responses. A double bounded dichotomous choice format was chosen because, although the NOAA panel report on CV (1993) [22] recommended the use of single bounded dichotomous choice as the only possibility of an “incentive-compatible choice”, a double bounded with a follow up question provides statistical efficiency and more precise welfare estimates [23]. Also, Bateman et al (2008) [24] introduced a Learning Design Contingent Valuation which assumed that repetition and experience in the valuation exercise reduce internal inconsistencies between the first and follow-up question. As choices are repeated and respondents gain familiarity with the decision environment, decisions become less random and more statistically efficient [25]. The questionnaire design also aimed at addressing coherent arbitrariness [26], starting point bias [27, 28] or sequential effects [29]. The questionnaire included an initial evaluation task with two different sequences and four double bound questions on WTP to reduce the risk of restenosis, and a second evaluation task with one double bound question on WTP to avoid restenosis. Finally, respondents answered a number of questions regarding their income, whether they have taken into account that they would have to pay for this, that they could not use that money to pay anything else, if they have the money they have accepted to pay and how would they obtain the money if they did not have it at that moment.

The aim of the questionnaire is twofold; first, it focuses on collecting data to obtain preferences for the DES and second, on obtaining data to elucidate preferences for a treatment with pills The second section of this questionnaire is analyzed here. The analysis of responses in the first section of the questionnaire provided evidence on respondents´ evaluation of the primary treatment of restenosis, DES, and on potential responses biases, such as scope effects [30], which would strengthen the validity of our results and overcome limitations of the WTP design [31].

Initially the participants are informed of the objectives and nature of the study. The interviewer provides information on the causes and symptoms of coronary artery occlusion and how it is usually addressed by the patient undergoing angioplasty to have a stent implanted. The angioplasty procedure is explained in detail and described as minimally invasive and the participants are informed that patients can usually walk unaided after about six hours, leave the hospital within 24 h and be fully recovered within a week. After the evaluation task on willingness to pay for a drug-eluting stent (DES), the questionnaire focuses on the evaluation task for avoiding angioplasty:

As has already been explained, when a fat plaque forms in the coronary artery, it can trigger a heart attack and the usual procedure is angioplasty. This procedure is not complicated, it does not require general anesthesia and patients are usually released from the hospital that very same day. However, patients need two or three days to recover and entering an operating theater is associated with discomfort.

Assume now that an alternative treatment is available, one that is equally effective as a Drug Eluting Stent and does not require surgery—namely, a treatment with pills option that would prevent one from needing to undergo surgery.

The interviewer explains that health outcomes, i.e., the reduction in the probability of restenosis, with the treatment with pills option and with angioplasty is the same. The only benefit of the treatment with pills option compared to a stent is avoidance of the angioplasty required to implant it.

For example:

- If one opts for the Drug Eluting Stent, the risk of repeated revascularization is 10% (10 cases out of 100).
- If one opts for the treatment with pills option, the risk of repeated revascularization is 10% (10 cases out of 100); as effective as a DES without one having to suffer the discomfort associated with surgery.

The participants are then asked if they fully understand the process and whether they have any questions. The interviewer repeats the information if necessary.

The participants then respond to a set of questions on their WTP for the treatment with pills option and for avoiding angioplasty. An initial question identifies the individual preferences for the treatment with pills option:

If you have to choose between undergoing surgery to have a DES implanted or being treated with pills, assuming both options are equally effective, which would you choose?

**Table:** 

Option A. Treatment with pills	1
Option B. Surgery	2 Give reasons for opting for surgery

Cost information is not provided at this point of the survey and the choice is made taking only the benefit of avoiding the operation into consideration. Individuals choosing the surgery option do not continue with WTP questionnaire after this point and are not included in the WTP calculation. Instead, they were posed a question on the reasons for their preference of the traditional (DES) versus a non –invasive treatment. Those who preferred the treatment with pills option were then asked to provide information on their WTP:

Assume now that you live in a country where Drug Eluting Stents are covered by the Social Security System (DES are free) but that there are some costs you have to pay associated with the treatment with pills option.

Please, look at the following amounts and tell us if you think you would or would not pay that amount. In this case, would you pay for treatment that avoids the need for surgery and is as efficient as a Drug Eluting Stent? To answer, please look at these amounts and indicate whether or not you would pay them:

**Table:** 

Option A. I would avoid surgery, choose the treatment with pills option, and pay X€	
Option B. I would opt for the Drug Eluting Stent and wouldn’t pay for the treatment with pills option	

The evaluation task consists of a choice scenario with an amount (referred to herein as “bid”) and three possible options, YES (I would pay), NO (I wouldn’t pay) or not providing an answer (N/A). If the respondent is willing to pay the initial bid (X), a second question follows with a higher price (*bid_up*). If the respondent refuses to pay the first bid, the second question includes a lower price (*bid_down*).

The default option for those not accepting the bid is the *status quo,* a percutaneous coronary intervention to implant a DES without any out-of-pocket payment. When the participant is willing to pay, they are reminded that 1) if they agree to pay that amount they cannot use that money for anything else, and 2) they do not need to consider the amounts they could only afford at that time as some costs are payable via credit or are paid gradually over time.

The values of the first bid are selected randomly from a set of bids. The first and follow-up bids are shown in euro in Table 2. The bids were tested in a sample survey with 100 observations in order to check whether the range was wide enough to reflect the true WTP curve.

**Table 2:** Bids (€)

*First bid*	*Follow-up bid*	
100	*bid*_*up*_	400
	*bid*_*down*_	30
400	*bid*_*up*_	900
	*bid*_*down*_	100
900	*bid*_*up*_	1500
	*bid*_*down*_	400
1500	*bid*_*up*_	3000
	*bid*_*down*_	900
3000	*bid*_*up*_	6000
	*bid*_*down*_	1500
6000	*bid*_*up*_	12,000
	*bid*_*down*_	3000
18,000	*bid*_*up*_	30,000
	*bid*_*down*_	12,000

Finally, information was gathered on the level of fear and anxiety associated with angioplasty, age, gender, schooling, occupation, family size, net disposable income, and a few notes on participants’ attitude and understanding.

### Double bounded dichotomous choice model

WTP responses were analyzed using a double bounded (DB) logit model [23]. In the empirical exercise the sequence of responses can be represented as follows. If the participant accepts to pay the first bid (q_1_ = Y) and the second bid (q_2_ = Y) the sequence is represented as YY. If the participant rejects to pay the first bid (q_1_ = N) and the second bid (q_2_ = N), the sequence is represented as NN. If the participant accepts to pay the first bid and rejects to pay the second, the sequence of responses is YN; finally, if the participant rejects to pay the first bid and accepts to pay the second bid, the sequence of responses is NY.

**Fig. 1:**
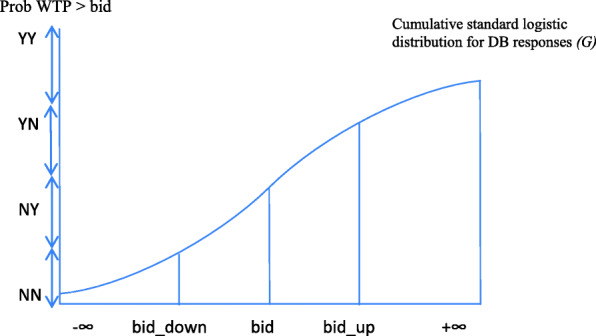
WTP intervals obtained from double-bounded questions

Participants accept the bid only if their WTP is higher, and reject it otherwise. Assuming that WTP is constant throughout the evaluation task, the sequence of responses provides an interval in which the true WTP falls (see Fig. 1).

Assuming that WTP for participant *i* (*WTP*_*i*_) is,

1 $$ WT{P}_i=\mu +{\varepsilon}_i $$

where *ε*_*i*_ is an error term following a logistic distribution with a mean of 0 and a variance of ${\tau}^2\frac{\pi^2}{3}$, being *τ* the scale parameter.

Under this framework, the probability of the sequence of responses is given by Hanemann et al. [23]:

$$ {\displaystyle \begin{array}{l}\left(\mathrm{i}.\right)\to \mathrm{q}1=\mathrm{Y},\mathrm{q}2=\mathrm{Y}\\ {}=\mathit{\Pr}\left({WTP}_i\ge {bid}_{up}\right)\\ {}\begin{array}{c}=\mathit{\Pr}\left(\mu +{\varepsilon}_i\ge {bid}_{up}\right)\\ {}\begin{array}{c}=\mathit{\Pr}\left({\varepsilon}_i\ge {bid}_{up}-\mu \right)\\ {}=1-G\left(\frac{bid_{up}-\mu }{\tau}\right)\end{array}\end{array}\end{array}} $$

$$ {\displaystyle \begin{array}{c}\left(\mathrm{ii}.\right)\to {\mathrm{q}}_1=\mathrm{N},{\mathrm{q}}_2=\mathrm{N}\\ {}\begin{array}{c}=\mathit{\Pr}\left({WTP}_i<{bid}_{down}\right)\\ {}=\mathit{\Pr}\left(\mu +{\varepsilon}_i<{bid}_{down}\right)\\ {}\begin{array}{c}=\mathit{\Pr}\left({\varepsilon}_i<{bid}_{down}-\mu \right)\\ {}=G\left(\frac{bid_{down-}\mu }{\tau}\right)\end{array}\end{array}\end{array}} $$

$$ {\displaystyle \begin{array}{c}\left(\mathrm{iii}.\right)\to {\mathrm{q}}_{\mathrm{i}}=\mathrm{Y},{\mathrm{q}}_2=\mathrm{N}\\ {}\begin{array}{c}=\Pr \left({ bi d}_1\le WTP<{ bi d}_{up}\right)\\ {}=\Pr \left({ bi d}_1-\mu +{\upvarepsilon}_i<{ bi d}_{up}\right)\\ {}\begin{array}{c}=\Pr \left(\frac{bi{d}_1-\mu }{\tau}\le \frac{\upvarepsilon_i}{\tau }<\frac{bi{d}_{up-\mu }}{\tau}\right)\\ {}=G\left(\frac{bi{d}_{up}-\mu }{\tau}\right)-G\left(\frac{bi{d}_1-\mu }{\tau}\right)\end{array}\end{array}\end{array}} $$

$$ {\displaystyle \begin{array}{c}\left(\mathrm{iv}.\right)\to {\mathrm{q}}_1=\mathrm{N},{\mathrm{q}}_2=\mathrm{Y}\\ {}=\mathit{\Pr}\left({bid}_{down}\le WTP<{bid}_1\right)\\ {}=\mathit{\Pr}\left({bid}_{down}\le \mu +{\varepsilon}_i<{bid}_1\right)\\ {}\begin{array}{c}=\mathit{\Pr}\left(\frac{bid_{down}-\mu }{\tau}\le \frac{\varepsilon_i}{\tau }<\frac{bid_1-\mu }{\tau}\right)\\ {}=G\left(\frac{bid_1-\mu }{\tau}\right)-G\left(\frac{bid_{down}-\mu }{\tau}\right)\end{array}\end{array}} $$

Estimates for $\gamma =-\frac{1}{\tau }$ and $\alpha =\frac{\mu }{\tau }$ are obtained by applying maximum-likelihood estimation. The log-likelihood function for this model is:

$$ \ln L=\sum \limits_{i=1}^N\left[{I}_i^{YY}\ln \left(1-G\left(\frac{b\mathrm{i}{d}_{up}-\mu }{\tau}\right)\right)\right.+ $$

$$ {I}_i^{NN}\ln {\displaystyle \begin{array}{c}\left(G\left(\frac{bi{d}_{down}-\mu }{\tau}\right)\right)+\\ {}\kern0.5em \end{array}} $$

$$ {I}_i^{YN}\ln \left(G\left(\frac{bi{d}_{up}-\mu }{\tau}\right)-G\left(\frac{bi{d}_1-\mu }{\tau}\right)\right) $$

$$ {I}_i^{NY}\ \left.\ln \left(G\left(\frac{bi{d}_1-\mu }{\tau}\right)-G\left(\frac{bi{d}_{down}-\mu }{\tau}\right)\right)\right] $$

And mean WTP is given by:

$$ E(WTP)=\mu =-\frac{\alpha }{\gamma } $$

In order to check for differences in WTP that could be explained by respondents´ socioeconomic differences or for the initial bid offered, we run two probit models, Model 1 to obtain coefficients for variables age, gender, years of study, employment and Model 2 that includes five dummies (k-1) corresponding to initial bids (k) that could be included in WTP estimations, as such,

$$ WTP=-\left({\beta}_0+{\beta}_1 age+{\beta}_2 gender+{\beta}_3 stud{y}_{years}+{\beta}_4 employment+{\beta}_5 bi{d}_{100}+{\beta}_6 bi{d}_{400}+{\beta}_7 bi{d}_{900}+{\beta}_8 bi{d}_{1500}+{\beta}_9 bi{d}_{3000}+{\beta}_{10} bi{d}_{6000}\right)/ bid $$

Coefficients and mean WTP were obtained in STATA.

## Results

A total of 1514 participants reached the second part of the questionnaire. 114 individuals preferred treating restenosis undergoing an angioplasty over the treatment with pill and were not willing to pay for this option. These individuals did not continue with the monetary evaluation of the treatment with pill. Specified reasons for refusing to pay for the pills included the idea of the operation being more effective, and fear of side and long-term effects of the pill treatment option. Observations for nine individuals that answered “N/A” to a bid or who did not provide other relevant information were not considered. The final sample size was 1391 individuals.

Respondents showed strong support for the treatment with pills. Responses to the initial question on preferences for treating restenosis with either angioplasty or by swallowing a pill are clear: 92.5% of the respondents opt for the treatment with pills. Response distribution according to gender, level of studies and employment status are presented in Tables 3 and 4.

**Table 3:** Response distribution socioeconomic characteristics of survey participants (% with-in group distribution)

		YES	NO
Gender	Female (*N* = 746)	91.69	8.31
	Male (*N* = 768)	93.23	6.77
Level of education	No studies (*N* = 88)	94.32	5.68
	Elementary and Middle School (*N* = 813)	92.49	7.51
	High School (*N* = 349)	89.97	10.03
	Bachelor, Master and PhD (248)	95.16	4.84
Employment Status	Unemployed (606)	91.91	8.09
	Employed (907)	92.83	7.17

**Table 4:** Response distribution and socioeconomic characteristics of survey participants (% intra-group distribution)

		YES	NO
Gender		*N* = 1400	*N* = 114
	Female	48.86	54.39
	Male	51.14	45.61
Level of education		*N* = 1385	*N* = 129
	No studies	5.95	4.39
	Elementary and Middle School	53.94	53.51
	High School	22.53	30.71
	Bachelor, Master and PhD	16.93	10.53
Employment Status		*N* = 1399	N = 114
	Unemployed	39.81	42.98
	Employed	60.19	57.02

### WTP for a treatment will pills to avoid angioplasty

Table 5 presents coefficient estimates, t-statistics and average WTP for the complete sample. Model coefficients behave as expected; bid coefficient is negative and all coefficients are statistically significant at 1 % level. Mean WTP for the treatment with pill is €5692.87 for the sample considered. This result is higher than the findings by Greenberg et al. [32] who obtained a WTP of $1162 to reduce all risk of restenosis (estimated as a 30% reduction) and by Guertin et al.*,* [33] who obtained a WTP of $2802 to reduce the probability of restenosis to 0. The population sample of these two studies are patients that have already undergone an angioplasty and do not include a societal perspective [34].

**Table 5:** DB model coefficients estimates and mean WTP estimates

Coefficient estimates (t-statistics)	
Constant	1.0539^***^(14.853)
Bid	−0.00018^***^(−28.334)
**WTP estimates**	
WTP (€)	5692.87
Standard Deviation	297.22
95% CI	(4867.61 6512.63)
Karl Pearson Coefficient of Dispersion	0.0522
Sample size	1391

^***^p < 0.01

Table 6 shows the results of the analysis including socioeconomic covariates (Model 1) that show higher mean WTP €6205.82. This Table also includes estimates for Model 2 with additional dummies for initial bids, WTP is €7370.59.

**Table 6:** Coefficients and mean WTP estimates

	Model 1	Model 2
Ind. Variables	Coefficients (95% CI) [Standard Error]	
Age	.0052822** (.0004496 .0101149) [.0024657]	.0051461** (0002551 .0100371) [.0024954]
Gender	.1013903 (−.0383144 .241095) [.0712792]	.0980641 (−.0434782 .2396063) [.0722168]
	.028903***	.0313319***
Study_years	(.0109181 .0468879) [.0091762]	(.0130473 .0496166) [.0093291]
Employment	.1719511** (.009848 .3340541) [.0827072]	.1700977** (.0067614 .3334341) [.0833364]
Bid_100		1.437932*** (.7110989 2.164766) [.3708403]
Bid_400		.5653523*** (.2590541 .8716505) [.1562775]
Bid_900		.3821457*** (.0933954 .670896) [.1473243]
Bid_1500		.0285884 (−.2522103 .309387) [.1432672]
Bid_3000		.0446755 (−.2118006 .3011517) [.1308576]
Bid_6000 Constant	−.173815 (−.5522919 .204662) [.193104]	0 (omitted)−.4759735 (−.916887 -.03506) [.22496]
**WTP estimates**		
WTP (€)	6205.826 (5289.2397122.414) [467.6551]	7370.596 (5652.0019089.19) [876.8501]
Z	13.27	9.36
N	1370	

Mean WTP is comparable to the average cost of a percutaneous angioplasty to implant DES in Spain, in 2009, which was estimated at an average cost of €5222.65, as specified in Table 7.

**Table 7:** Cost of percutaneous angioplasty to implant DES (Spain, 2009. Red Española de Costes Hospitalarios (RECH), https://www.rechosp.org/)

Mean cost (€)	5222.65
Standard Deviation	2947.85
95% CI	(5004.27 5441.03)
Karl Pearson Coefficient of Dispersion	0.5644
Sample size	700

Socioeconomic characteristics such as age, years of study and being employed are significant to explain bid acceptance and differences in WTP estimations.

## Discussion

In this article two issues are introduced, why it is important to examine process utility and how such process utility might be estimated empirically. First, our results provide evidence of the existence of process utility in line with previous findings [1, 21, 35, 36]. People value avoiding angioplasty, which is a short, uncomplicated surgical procedure. Second, our CV study elicited a significant WTP for a benefit that in terms of QALYs is modest. Estimates of QALYs lost in angioplasty range from 0.0035 to 0.08 QALYs [37–46]. Ploegmakers et al.*,* [47] find no disutility in terms of QALYs related to angioplasty. These differences in QALY’s measurements depend on different factors, such as the utility over the follow-up procedure and the length of the timeframe used in the analysis. Cohen and Baim (1995) [48] found that an intervention with initial stenting would save an additional 0.03 QALY (2 healthy weeks) with respect to standard angioplasty whereas angioplasty with stenting for restenosis would only save an additional 0.01 QALY in comparison with standard angioplasty [37]. These numbers emphasise the constraints associated with using QALY analysis to assess the utility gains associated with the avoidance of restenosis.

For our purpose, we have selected the estimation of QALY that seems representative of what our respondents are evaluating: a treatment that would avoid angioplasty. Therefore, the value of 0.0056 QALYs lost in angioplasty from Bagust et al.*,* [38] comply with characteristics that respondents have evaluated.

This WTP estimate imply a monetary value per QALY that greatly exceed the cost per QALY thresholds established in different countries to consider health programs as beneficial to society: €25,000 in Spain [49], £25,000–£35,000/QALY in the UK [50], €20,000–€80,000 (for severe diseases)/QALY in The Netherlands [51], $60,000–$75,000 in Japan [52] and $50,000/QALY and CAD$50,000/QALY in the US and Canada, respectively [53].

Therefore, these results reflect how different methodologies for HTA may lead to different conclusions in the case in question. From the ICER perspective, the cost that would make the treatment with pills option cost-effective, using a threshold of €40,000/QALY, would be €224. However, a cost-benefit approach could support health programs even with a higher cost.

Finally, it seems that WTP methodology is able to capture outcome and process factors related to angioplasty as our WTP estimations are non significantly different for the value of the benefit individuals are evaluating. It seems that individuals are willing to pay as much as the cost of the surgery that they are avoiding. Socioeconomic characteristics such as age and years of study, and lower initial bids might explain WTP increases.

## Conclusions

Findings are robust since 1) respondents have evaluated an hypothetical treatment that would avoid discomfort and/or fear of surgery and that is exactly what we have estimated in our results; 2) respondents have previously evaluated an alternative option, what they would have to go through in the case they opt for an angioplasty to implant a DES, we expect our results to be “less erratic” that in surveys were respondents are only asked to value one type of intervention, as hypothesized by Donaldson and Shackley [1].

Since both outcome and process characteristics of the intervention are clear for respondents the contribution of WTP to detect process utility will also be clear. WTP estimation is similar to the average cost of an angioplasty and coefficient of deviation is much shorter. The cost of angioplasty depends on the severity of the condition and factors such as the number of the stents implanted or days in hospital, which explains the higher dispersion in estimations. Respondents were asked to value a general, average, procedure, and their WTP is not different to average cost. WTP lower coefficient of dispersion demonstrate low variability within observations, leading us to conclude that respondents valued what they were asked to value.

The WTP approach must be considered as a genuine alternative to QALY approaches to value process utility. Notwithstanding, more research comparing the different approaches in the cases where process utility is involved are needed. Studies obtaining QALY gains and WTP estimates for process utility from the same sample could be very helpful to compare their suitability in these cases.

Our proposal is not free of critics and limitations itself. For example, it requires empirical work on willingness to pay to obtain the utility value of health. It only obtains one value for the alternative treatment to restenosis and it is difficult to compare across health states.

## Data Availability

All data is available upon request.
